# Would You like a New Nose?

**Published:** 1890-03

**Authors:** 


### WOULD YOU LIKE A NEW NOSE?

### A BOSTON SURGEON WHO MAKES NASAL ORGANS OF ANY SHAPE TO ORDER.

There can be no greater deformity than the loss of one's nose. Any kind of an organ, whether pug, turned up or flat, is preferable to an entire absence of one.

In olden times, men and women who were so unfortunate as to lose this member, unless wealthy, had to get on as best they might, but the individuals of great substance could provide themselves with artificial ones of ivory, gold or silver, usually enameled and of flesh color. These substitutes were, of course, awkward and uncomfortable, but the victims endured them because they concealed, or were thought to conceal, their loss.

In latter days surgery came to the aid of the noseless, and what is called plastic surgery. or Talicotean operations are performed. These consist in taking adjacent pieces of skin, and twisting and turning them to unite with the freshly cut edges of the remnant of the original nose. Plugs of cotton and quills through which to breathe from the nostrils, and the result is a new nose, but of a putty-like appearance, as it has no bridge. Such a nose, as everybodv knows, is better than no nose at all, but the new ones of surgery were by no means beautiful.

But a wonderful revolution in the restoring of that most useful and ornamental organ has within a month or two taken place in this city, which, so far as is known, is the first of the kind in the United States. Dr. H. D. Burrell, surgeon of the City and Carney hospitals, it is who has made the discovery in the way of furnishing a bridge for the nose.

At the time spoken of above, an otherwise fine looking woman from one of the thriving towns of the state, with a not at all pleasant apology for a nose, applied to Dr. Burrell for relief. Her deformity was such that she was compelled to live a most secluded life, rarely going out, save at night for needed exercise.

It occurred to Dr. Burrell that an improvement might be made on previous operations, and that, by adding a properly shaped piece of bone from a living animal, a natural looking nose could be made. An appointment was given the woman at Carney hospital and an operation was performed by cutting out the side of the mutilated member and turning it completely over.

A young chicken was then killed, and a piece of the breast bone of proper shape united to the root of the nose on the skull by silver wires.

Then the flesh of the old nose was properly stretched over the bone and secured by ligatures. Tamkins of cotton were put in to give the nostrils the proper shape.

The new bone became properly united with its attachment, the wound healed, and the operation, thus far, is a perfect success. The patient has now a fine Roman nose, and the only scar is a nearly imperceptible line at one side of the newly-made organ.—*Boston Herald.*

